# Coagulation factor VIII, white matter hyperintensities and cognitive function: Results from the Cardiovascular Health Study

**DOI:** 10.1371/journal.pone.0242062

**Published:** 2020-11-16

**Authors:** Jessica L. Rohmann, W. T. Longstreth, Mary Cushman, Annette L. Fitzpatrick, Susan R. Heckbert, Kenneth Rice, Frits R. Rosendaal, Colleen M. Sitlani, Bruce M. Psaty, Bob Siegerink

**Affiliations:** 1 Charité –Universitätsmedizin Berlin, Center for Stroke Research Berlin, Berlin, Germany; 2 Charité –Universitätsmedizin Berlin, Institute of Public Health, Berlin, Germany; 3 Department of Neurology, University of Washington, Seattle, WA, United States of America; 4 Department of Epidemiology, University of Washington, Seattle, WA, United States of America; 5 Department of Medicine, Larner College of Medicine at the University of Vermont, Burlington, VT, United States of America; 6 Department of Family Medicine, University of Washington, Seattle, WA, United States of America; 7 Department of Global Health, University of Washington, Seattle, WA, United States of America; 8 Cardiovascular Health Research Unit, University of Washington, Seattle, WA, United States of America; 9 Department of Biostatistics, University of Washington, Seattle, WA, United States of America; 10 Department of Clinical Epidemiology, Leiden University Medical Center, Leiden, The Netherlands; 11 Department of Medicine, University of Washington, Seattle, WA, United States of America; 12 Department of Health Services, University of Washington, Seattle, WA, United States of America; 13 Kaiser Permanente Washington Health Research Institute, Seattle, WA, United States of America; McLean Hospital, UNITED STATES

## Abstract

**Objective:**

To investigate the relationship between high FVIII clotting activity (FVIII:C), MRI-defined white matter hyperintensities (WMH) and cognitive function over time.

**Methods:**

Data from the population-based Cardiovascular Health Study (n = 5,888, aged ≥65) were used. FVIII:C was measured in blood samples taken at baseline. WMH burden was assessed on two cranial MRI scans taken roughly 5 years apart. Cognitive function was assessed annually using the Modified Mini-Mental State Examination (3MSE) and Digit Symbol Substitution Test (DSST). We used ordinal logistic regression models adjusted for demographic and cardiovascular factors in cross-sectional and longitudinal WMH analyses, and adjusted linear regression and linear mixed models in the analyses of cognitive function.

**Results:**

After adjustment for confounding, higher levels of FVIII:C were not strongly associated with the burden of WMH on the initial MRI scan (OR>p75 = 1.20, 95% CI 0.99–1.45; N = 2,735) nor with WMH burden worsening over time (OR>p75 = 1.18, 95% CI 0.87–1.59; N = 1,527). High FVIII:C showed no strong association with cognitive scores cross-sectionally (3MSE>p75 β = -0.06, 95%CI -0.45 to 0.32, N = 4,005; DSST>p75 β = -0.69, 95%CI -1.52 to 0.13, N = 3,954) or over time (3MSE>p75 β = -0.07,95% CI -0.58 to 0.44, N = 2,764; DSST>p75 β = -0.22, 95% CI -0.97 to 0.53, N = 2,306) after confounding adjustment.

**Interpretation:**

The results from this cohort study of older adult participants indicate no strong relationships between higher FVIII:C levels and WMH burden or cognitive function in cross-sectional and longitudinal analyses.

## Introduction

White matter hyperintensities (WMH) on MRI are common among older adults. According to a Dutch community-based study, the prevalence of WMH in healthy volunteers aged between 60 and 90 years was estimated to be 95%, and both prevalence and severity were found to increase with age [1]. In addition to being associated with traditional cardiovascular risk factors, changes in brain morphology, including WMH development, have been implicated in cognitive decline, incident cognitive impairment, and the development of dementia [2–6].

Coagulation factor FVIII (FVIII) levels generally increase with age [2]. Acute elevation of FVIII is known to occur during the acute phase of stroke as part of the inflammatory response [3]. A dose-dependent relationship between FVIII levels and the occurrence of thrombotic events (including overt ischemic stroke) has also been observed [4–8]. Further studies have linked FVIII, a potential therapeutic target, with dementia risk [9,10] as well as risk for cognitive impairment in a study of men [11]. Previous research in the US-based Cardiovascular Health Study (CHS) found an association between higher FVIII and incident cardiovascular disease, stroke, and death in the older general study population [12], but these studies did not investigate covert WMH or cognitive decline.

It remains unclear whether FVIII may contribute to the severity of covert WMH burden and worsening over time. It is also unknown whether high FVIII levels contribute to cognitive decline in this population, a process that may, in turn, be mediated by WMH. The present study aims to probe these relationships to better understand the role of FVIII, if any, in the pathways leading to cognitive decline in both cross-sectional and longitudinal settings, using data from a large, population-based cohort.

## Methods

### Participants and design

**Fig 1:**
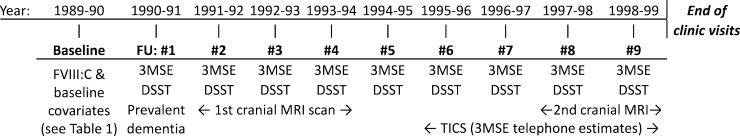
Timeline of CHS data collection. Measurements collected at baseline and at each follow-up are listed per year. Abbreviations: FU, follow-up; FVIII:C, coagulation factor VIII activity; 3MSE, Modified Mini-Mental State Examination; DSST, Digit Symbol Substitution Test; MRI, magnetic resonance imaging, TICS, Telephone Interview for Cognitive Status.

In this cohort study, we used data from the Cardiovascular Health Study (CHS) in all analyses. The design of the full longitudinal, population-based CHS, which aimed to assess risk factors for cardiovascular disease is described in detail elsewhere [13]. Briefly, the original CHS cohort included adults aged 65 and older recruited from four United States communities using Medicare eligibility lists. The first cohort of participants (N = 5,201) were enrolled in 1989 and 1990. A second cohort oversampling African-Americans (n = 687) was enrolled in 1992 and 1993; however, since FVIII levels were not measured in this group, the second cohort could not be included in our analyses. As illustrated in Fig 1, participants were prospectively followed for 9 years after the baseline visit and completed yearly clinic visits or phone interviews. An overview of all relevant variables used in our study and a timeline of their acquisition in the CHS is shown in Fig 1.

### Baseline assessment

The exposure variable of interest, FVIII clotting activity (FVIII:C), was measured in blood samples of 5,112 participants taken at baseline. Clotting activity was assayed using the Coag-a-mate X2 instrument with WHO standards, and activity units are expressed as percentages of normal pooled plasma [14]. The mean coefficients of variation of two control pools were 9% and 10%.

Other relevant variables measured at baseline included self-reported age, sex, and ethnicity. Participants were also asked to provide information about their highest level of completed education and information regarding smoking status and alcohol consumption. Smoking status was categorized as never, former, or current. Alcohol consumption was classified as never, occasionally, or frequently, which was defined as consuming more than 7 drinks per week, on average.

We considered other measurements taken at the baseline clinic visits as markers for cardiovascular risk. Participants’ measured weight (kg) and squared height (m^2^) were used to compute body mass index (BMI). Individuals presenting with ≥140 mmHg (systolic) or ≥90 mmHg (diastolic) seated blood pressure at baseline were classified as ‘hypertensive’ in addition to those who both reported a history of hypertension and were taking antihypertensive medication at baseline. High density lipoprotein cholesterol (HDL, mg/dL) and adjusted low-density lipoprotein cholesterol, (LDL, mg/dL) were measured from blood samples and recorded as continuous variables. Furthermore, participants’ fasting plasma glucose levels were used to determine diabetes status according to current American Diabetes Association guidelines as ‘normal’(<100mg/dL), ‘impaired fasting glucose’ (100-125mg/dL), or ‘diabetic’ (≥126 or reported taking insulin or oral hypoglycemics). Maximum common carotid intima-media thickness (CIMT) and maximum internal CIMT were defined as the mean of maximum wall thickness measurements made during all scans using ultrasonography used as indicators of carotid atherosclerosis [15]. Further relevant blood biomarker measurements included C reactive protein (CRP, mg/L) and fibrinogen (mg/dL). In addition to information on whether the participant had a history of stroke or transient ischemic attack (TIA) at baseline, incident stroke or incident TIA events occurring during CHS follow-up were also recorded [16].

### Ascertainment of outcomes

Cranial MRI scans were performed in two waves over several years. The initial scan occurred during the 2nd through 4th follow-up visits (1991–1994), and the follow-up scan during the 8th and 9th follow-up visits (1997–1999), allowing for the assessment of changes in brain morphology in CHS participants over an average time interval of 5 years [17,18]. Areas of hyperintensities in the periventricular and subcortical regions as observed on standardized sagittal axial-spin density/T2-weighted cranial MRI images were used to quantify WMH burden, one of our outcomes of interest [19]. The WMH burden apparent on each scan was evaluated by experienced neuroradiologists at a central CHS Reading Center using a 10-point white matter grade (WMG) scale, ranging from 0 (no lesions) to 9 (most lesions) based on a library of templates [18]. In additional, the initial and follow-up MRI scans were read side-by-side to determine any worsening of the WMG between the two scans [17].

Cognitive ability was measured using multiple assessment tools. The first, the 100-point Modified Mini-Mental State Examination (3MSE) [20], was introduced during the first follow-up wave (1990–91) and administered annually thereafter during in-person clinic visits. Missing 3MSE scores could be estimated from the Telephone Interview for Cognitive Status (TICS) scores, when these data were available. TICS were first introduced during the sixth follow-up (1995–96), and these estimates have previously been confirmed to be reliable substitutions for 3MSE scores [21].

To provide an indication of the robustness of our results, we included an additional assessment of cognitive function as a second outcome: the Digit Symbol Substitution Test (DSST). This timed, 90-second test measures both attention and processing speed. The DSST overcomes the known challenge of the ceiling effect of the 3MSE [22]. The DSST was administered at baseline (1989–90) and annually thereafter during in-person clinic visits. For consistency between the two outcome measures, we considered only 3MSE scores (or the imputed TICS estimates thereof) and DSST scores measured during the 1st through 9th follow-up visits as continuous outcomes in our longitudinal analyses.

### Inclusion/Exclusion criteria

In addition to the general inclusion and exclusion criteria of the CHS [13], we additionally excluded from our analyses all participants with missing FVIII:C measurements or with a history of one or more of the following at baseline: clinical stroke, TIA, prevalent dementia, and/or low cognitive function. Low cognitive function was defined as scoring less than 78 on the first 3MSE measurement, an established cutoff used in other CHS cognition studies [23], or in the lowest 10% of all scores on the first included DSST. Prevalent dementia and cognitive function (as measured by 3MSE) were first assessed in 1990–91.

### Statistical analyses FVIII:C categorization

For our primary analyses, participants were categorized according to their FVIII activity levels (FVIII:C). High (>75th percentile) and low (≤25th percentile) FVIII:C levels were compared to the middle reference interval between the 25th and 75th percentiles, based on our *a priori* analysis plan. In secondary analyses, FVIII:C were analyzed continuously as normalized variables by dividing FVIII:C by the standard deviation (SD) of all FVIII:C measurements. To explore possible dose response, we used quintiles to group FVIII:C using the middle fifth as a reference. As a sensitivity analysis to assess robustness of our findings, we performed an additional categorization of FVIII:C. High (>90th percentile) and low (≤10th percentile) FVIII:C levels were compared to participants with FVIII:C in the 10th– 90th percentile interval (reference).

### White matter hyperintensity burden at initial cranial MRI scan: Cross-sectional analyses

Cross-sectional analyses were conducted to determine whether FVIII:C levels were associated with WMH burden on the initial MRI scan (see Fig 1) in all participants having initial scan results. For this analysis, the outcome variable, WMH burden, was grouped by grade into three groups (0–1, 2–3, 4–9). Differences in mean FVIII:C between WMG groups were assessed using an ANOVA.

We then used ordinal logistic regression to probe the relationship between FVIII:C and WMH burden using the gologit2 command in Stata, which can relax the proportional odds assumption as needed for specific explanatory variables (this is known as fitting a partial proportional odds model) [24,25]. In addition to an unadjusted model (model 1), we provide two additional models conditioning for potential confounding variables: model 2: adjusted for *a priori* selected demographic and socioeconomic factors including age, sex, ethnicity, and education level; and model 3: additionally adjusting for *a priori* selected lifestyle and cardiovascular risk factors: smoking, alcohol use, BMI, hypertension, diabetes, HDL and LDL cholesterol, fibrinogen, CRP, maximum common and internal CIMT, and the occurrence of a TIA or stroke event during the follow-up period prior to the initial MRI scan, as defined previously. Logarithmic transformations were used for variables with right-skewed distributions including both CIMT measurements and CRP. Since incident TIA or stroke events could be mediators for the exposure-outcome relationship, we conducted an additional sensitivity analysis in which this variable was omitted. We report ordinal odds ratios with corresponding 95% confidence intervals (95% CI) for each model.

### Worsening of white matter grade between MRI scans: Longitudinal analyses

We report odds ratios with corresponding 95% CI from ordinal logistic regression models to quantify the worsening in WMG between the two MRI scans taken on average 5 years apart. Thus, only participants who underwent both MRI scans were included in these analyses. Change between scans was *a priori* categorized as no worsening in WMG, a worsening of 1 grade, or a worsening of 2 or more grades. The analyses were additionally adjusted for the elapsed time between participants’ initial and follow-up MRI scans but not for baseline WML grade, as WML grade changes were likely to precede our baseline assessment and such adjustment is thus likely to introduce bias [26].

### FVIII:C and cognitive function: Cross-sectional and longitudinal analyses

We used linear regression models to obtain estimates of the effect of baseline FVIII:C (β and 95% CIs) on 3MSE scores measured at the first follow-up in three adjusted models as described above. We then used linear mixed models with random intercepts for each individual to investigate the relationship between FVIII:C and cognitive function over time, as measured by serial 3MSE scores. These models handle missing data and the dependent nature of the repeated measurements within individuals. As a secondary analysis, we repeated both cross-sectional and longitudinal analyses using DSST scores instead of 3MSE. Effect estimates and 95% CIs are reported for the 3 models, as well as the model variance between individuals.

### Standard protocol approvals, registrations and patient consents

Informed written consent was obtained from all CHS participants at entry into the study and at periodic intervals during follow-up. Institutional review boards at each CHS center approved the study. The IRB at the Charité - Universitätsmedizin Berlin also approved this secondary data analysis, and data was transferred in a fully anonymized form for analysis.

## Results

### Description of study population

**Fig 2:**
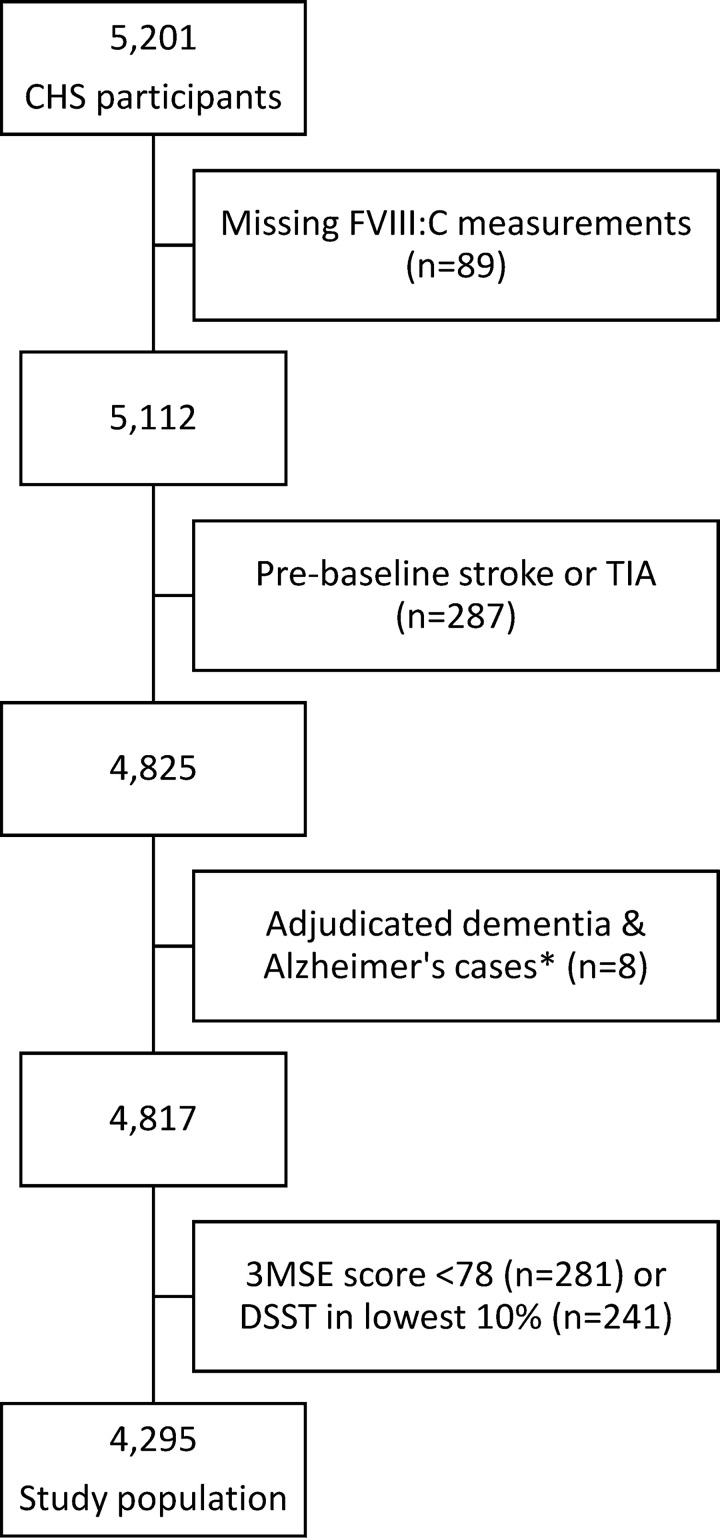
Study inclusion flow chart. Dementia was first adjudicated in 1990–91. DSST and 3MSE scores from that study year were also used to determine participant inclusion for this study. Abbreviations: FVIII:C, coagulation factor VIII activity; FU, follow-up; 3MSE, Modified Mini-Mental State Examination; DSST, Digit Symbol Substitution Test; MRI, magnetic resonance imaging; TICS, Telephone Interview for Cognitive Status; TIA, transient ischemic attack.

After applying our inclusion and exclusion criteria as shown in the participant flowchart (Fig 2), our study population consisted of 4,295 participants whose characteristics are summarized in Table 1. The study participants had a mean age of 72.3 years, 59.3% were female, and 96.0% were white. In total, 45.5% of included participants were hypertensive and 13.9% were diabetic.

**Table 1:** Baseline characteristics of study population.

Characteristic (n = 4,295)	
**Mean age (SD), years**	72.3 (± 5.3)
**Female sex, N (%)**	2,549 (59.3%)
**White, N (%)**	4,124 (96.0%)
**Education, N (%)**^**a**^	
< Grade 12	986 (23.0%)
Completed high school/GED	1,296 (30.2%)
Vocational or some college	1,045 (24.3%)
Graduate degree/professional	960 (22.4%)
**BMI (SD), kg/m**^**2**^	26.4 (± 4.5)
**Hypertension, N (%)**	1,950 (45.4%)
**Cholesterol (SD), mg/dL**	
Total	212.3 (± 39.0)
HDL	54.4 (± 15.9)
LDL	130.4 (± 35.4)
**Diabetes, N (%)**	
None	3,101 (72.2%)
Impaired fasting glucose	592 (13.8%)
Known or new	598 (13.9%)
**Smoking, N (%)**	
Never	1,971 (45.9%)
Former	1,841 (42.9%)
Current	481 (11.2%)
**Alcohol use, N (%)**	
Never	1,961 (45.7%)
Occasional	1,729 (40.3%)
Frequent	591 (13.8%)
**FVIII:C (IQR), %**^**b**^	116 (95–141)
**CRP (IQR), mg/L**	2.4 (1.2–4.2)
**Fibrinogen (IQR), mg/dL**	311 (270.0–361)
**Max Common CIMT (IQR), mm**	0.96 (0.86–1.08)
**Max Internal CIMT (IQR), mm**	1.33 (0.90–1.90)
**3MSE score (IQR)**^**c**^	94 (89–97)
**DSST score (IQR)**^**d**^	42 (35–51)

Abbreviations: GED = General Educational Development (high school equivalency diploma); BMI = body mass index; HDL = high-density lipoprotein; LDL = low-density lipoprotein; FVIII:C = coagulation factor VIII activity; CRP = C-reactive protein; CIMT = carotid intima-media thickness; 3MSE = Modified Mini-Mental State examination; DSST = Digit Symbol Substitution Test; IQR = interquartile range.

^a^ Owing to missing data, percentages may not total 100. All variables have <2% missing values except 3MSE (6.8%), and DSST (7.9%).

^b^ percentage of normal pooled plasma

^c^ out of 100 possible points, measured during 1991–92 study year

^d^ out of 90 possible points, measured during 1991–92 study year

### FVIII:C and white matter grade

Of the 2,735 participants who underwent the first MRI scan, mean FVIII:C differed among WMG groups (p = 0.001). Participants with the highest burden of WMH on this scan (grades 4–9) had higher mean FVIII:C (121.2) than participants with WMG of 2–3 (120.5) or WMG 0–1 (115.3) (see Table 2). Ordinal analyses using three groups of WMG (0–1, 2–3, and 4–9) revealed a weak association between high FVIII:C (>p75)–compared to normal levels–and WMG (model 3 OR = 1.20, 95% CI 0.99–1.45) in participants with initial MRI scan results (Table 2). Furthermore, each increase in FVIII:C by one standard deviation (36 units) was not associated with substantially worse WMG at the initial MRI scan after full adjustment (model 3 OR = 1.08, 95% CI 0.99–1.17).

**Table 2:** FVIII:C and burden of white matter hyperintensities on initial cranial MRI scan (cross-sectional).

	WMG 0–1 (n = 995)	WMG 2–3 (n = 1,371)	WMG 4–9 (n = 369)	OR1^b^	95% CI	OR2^c^	95% CI	OR3^d^	95% CI
**FVIII:C**^**a**^ **groups**									
≤ p25	285	337	97	1.06	(0.89–1.26)	1.03	(0.86–1.23)	1.03	(0.85–1.23)
p25-p75	522	690	182	1	ref	1	ref	1	ref
> p75	188	344	90	1.37	(1.11–1.68)	1.22	(1.02–1.46)	1.20	(0.99–1.45)
**Continuous**									
per SD increase of FVIII:C^e^				1.15	(1.07–1.24)	1.09	(1.01–1.17)	1.08	(0.99–1.17)

Abbreviations: FVIII:C = coagulation factor VIII activity; WMG = white matter grade; MRI = magnetic resonance imaging; OR = odds ratio; CI = confidence interval; p25 = 25th-percentile; p75 = 75th-percentile; ref = reference category; SD = standard deviation

^a^ percentage of normal pooled plasma

^b^ unadjusted model

^c^ model adjusted for demographic risk factors (age, gender, ethnicity, education level)

^d^ model additionally adjusted for cardiovascular risk factors (hypertension, smoking status, diabetes, alcohol use, BMI, HDL cholesterol, LDL cholesterol, fibrinogen, log-transformed C-reactive protein, log-transformed maximum common carotid intima-media thickness, log-transformed maximum internal carotid intima-media thickness, and occurrence of stroke or TIA prior to initial MRI scan)

^e^ one standard deviation increase in FVIII:C corresponds to 36 units

We report results from longitudinal analyses in Table 3. Considering worsening of WMG between the two scans, after full adjustment for potential confounding factors, the ordinal logistic regression analysis revealed no meaningful association between high FVIII:C (>p75) and the degree of worsening over the time period between scans (model 3 OR = 1.18, 95% CI 0.87–1.59).

**Table 3:** FVIII:C and white matter hyperintensity grade worsening on MRI scans over 5 years; results from ordinal logistic regression models.

	No change (n = 1,098)	1 grade worse (n = 367)	2+ grades worse (n = 62)	OR1^b^	95% CI	OR2^c^	95% CI	OR3^d^	95% CI
**FVIII:C**^**a**^ **groups**									
≤ p25	318	99	20	1.02	(0.78–1.33)	1.03	(0.79–1.35)	1.01	(0.76–1.33)
p25-p75	552	185	25	1	ref	1	ref	1	ref
> p75	228	83	17	1.16	(0.88–1.55)	1.15	(0.87–1.54)	1.18	(0.87–1.59)
**Continuous**									
per SD increase of FVIII:C^e^				1.05	(0.93–1.18)	1.04	(0.93–1.18)	1.07	(0.94–1.22)

Abbreviations: FVIII:C = coagulation factor VIII activity; WMG = white matter grade; MRI = magnetic resonance imaging; OR = odds ratio; CI = confidence interval; p25 = 25th-percentile; p75 = 75th-percentile; ref = reference category; SD = standard deviation

^a^ percentage of normal pooled plasma

^b^ model adjusted for time interval (in years) between MRI scans

^c^ model additionally adjusted for demographic risk factors (age, gender, ethnicity, education level)

^d^ model additionally adjusted for cardiovascular risk factors (hypertension, smoking status, diabetes, alcohol use, BMI, HDL cholesterol, LDL cholesterol, fibrinogen, log-transformed C-reactive protein, log-transformed maximum common carotid intima-media thickness, log-transformed maximum internal carotid intima-media thickness, and occurrence of stroke or TIA during follow-up period before second MRI scan

^e^ one standard deviation increase in FVIII:C corresponds to 36.3 units

Similar findings were observed in secondary analyses considering per SD increase in FVIII:C as the exposure (model 3 OR = 1.07, 95% CI 0.94–1.22).

### FVIII:C and cognitive function

Table 4 shows results from cross-sectional analyses using 3MSE (n = 4,005) and DSST (n = 3,954) scores from study year 1991–92 (first follow-up) as outcomes. Though a small association between high FVIII:C (>p75) and lower 3MSE scores was observed in the crude linear regression model (β_1_ = -0.55; 95% CI -0.95 to -0.15), this association disappeared after full confounding adjustment (β_3_ = -0.06, 95% CI -0.45 to 0.32). The unadjusted association with DSST score (β_1_ = -2.05, 95% CI -2.94 to -1.16), was also greatly reduced after full adjustment (β_3_ = -0.69, 95% CI -1.52 to 0.13). For the DSST scores, we observed a slight dose response in the crude quintile assessment. Per SD of FVIII:C, we also found no association between high FVIII:C and lower 3MSE or DSST scores in the fully adjusted models (Table 4).

**Table 4:** FVIII:C and cognitive test scores: Cross-sectional results using linear regression.

**3MSE scores (100 points maximum), study year 1991–92 (n = 4,005)**						
	**β**_**1**_ ^**b**^	**95% CI**	**β**_**2**_ ^**c**^	**95% CI**	**β**_**3**_ ^**d**^	**95% CI**
**FVIII:C**^**a**^ **percentile groups**						
≤ p25	0.61	(0.22 to 1.00)	0.33	(-0.03 to 0.69)	0.33	(-0.04 to 0.71)
p25-p75	0	ref	0	ref	0	ref
> p75	-0.55	(-0.95 to -0.15)	-0.16	(-0.53 to 0.21)	-0.06	(-0.45 to 0.32)
**Continuous**						
per SD increase of FVIII:C^e^	-0.40	(-0.56 to -0.23)	-0.16	(-0.31 to -0.01)	-0.14	(-0.30 to 0.03)
**FVIII:C quintile groups**						
Q1 (low)	0.22	(-0.24 to 0.68)	0.22	(-0.24 to 0.68)	0.22	(-0.25 to 0.69)
Q2	-0.24	(-0.70 to 0.22)	-0.24	(-0.70 to 0.22)	-0.25	(-0.72 to 0.22)
Q3	0	ref	0	ref	0	ref
Q4	-0.39	(-0.86 to 0.07)	-0.39	(-0.86 to 0.07)	-0.34	(-0.82 to 0.13)
Q5 (high)	-0.28	(-0.75 to 0.19)	-0.28	(-0.75 to 0.19)	-0.18	(-0.67 to 0.31)
**DSST scores (90 points maximum), study year 1991–92 (n = 3,954)**						
	**β**_**1**_	**95% CI**	**β**_**2**_	**95% CI**	**β**_**3**_	**95% CI**
**FVIII:C percentile groups**						
≤ p25	1.58	(0.71 to 2.44)	0.90	(0.13 to 1.68)	0.85	(0.06 to 1.64)
p25-p75	0	ref	0	ref	0	ref
> p75	-2.05	(-2.94 to -1.16)	-1.01	(-1.81 to -0.22)	-0.69	(-1.52 to 0.13)
**Continuous**						
per SD increase of FVIII:C^e^	-1.16	(-1.53 to -0.80)	-0.54	(-0.87 to -0.21)	-0.37	(-0.72 to -0.02)
**FVIII:C quintile groups**						
Q1 (low)	1.37	(0.26 to 2.48)	0.66	(-0.33 to 1.65)	0.53	(-0.48 to 1.53)
Q2	0.86	(-0.26 to 1.97)	0.57	(-0.43 to 1.56)	0.47	(-0.54 to 1.47)
Q3	0	ref	0	ref	0	ref
Q4	-0.86	(-1.99 to 0.27)	-0.21	(-1.21 to 0.80)	-0.12	(-1.14 to 0.89)
Q5 (high)	-1.84	(-2.98 to -0.70)	-0.80	(-1.81 to 0.22)	-0.42	(-1.46 to 0.63)

Abbreviations: FVIII:C = coagulation factor VIII activity; 3MSE = Modified mini-mental state examination; DSST = Digit Symbol Substitution Test; CI = confidence interval; p25 = 25th-percentile; p75 = 75th-percentile; ref = reference category; SD = standard deviation.

β coefficients were calculated using linear regression in three models.

^a^ percentage of normal pooled plasma

^b^ unadjusted model

^c^ model adjusted for demographic risk factors (age, gender, ethnicity, education level)

^d^ model additionally adjusted for cardiovascular risk factors (hypertension, smoking status, diabetes, alcohol use, BMI, HDL cholesterol, LDL cholesterol, fibrinogen, log-transformed C-reactive protein, log-transformed maximum common carotid intima-media thickness, and log-transformed maximum internal carotid intima-media thickness).

^e^ one standard deviation increase in FVIII:C corresponds to 36.3 units

Linear mixed-effects regression with random intercepts was then used to investigate whether FVIII:C measured at baseline were associated with cognitive ability over time (Table 5). The numbers of participants with available measurements in the final year of follow-up were N = 2,764 (3MSE or TICS estimate) and N = 2,306 (DSST). As was observed in the cross-sectional setting, having high (>p75) FVIII:C was not associated with 3MSE over the course of study follow-up in the fully adjusted models (β_3_ = -0.07, 95% CI -0.58 to 0.44; β_3_ per SD of FVIII:C = 0.15, 95% CI -0.06 to 0.37). Furthermore, no clinically relevant association was observed between high FVIII:C and change in DSST score over time, after adjustment (β_3_ = -0.22, 95% CI -0.97 to 0.53; β_3_ per SD of FVIII:C = -0.11, 95% CI -0.43 to 0.22).

**Table 5:** FVIII:C and cognitive test scores: Longitudinal results using linear mixed-effects regression.

**3MSE scores (100 points maximum)**						
	**β**_**1**_ ^**b**^	**95% CI**	**β**_**2**_ ^**c**^	**95% CI**	**β**_**3**_ ^**d**^	**95% CI**
**FVIII:C**^**a**^ **percentile groups**						
≤ p25	0.29	(-0.25 to 0.83)	-0.27	(-0.76 to 0.21)	-0.40	(-0.89 to 0.09)
p25-p75	0	ref	0	ref	0	ref
> p75	-1.09	(-1.64 to -0.54)	-0.34	(-0.83 to 0.15)	-0.07	(-0.58 to 0.44)
***Model variance*** ^**e**^	*46*.*7*	*(44*.*4 to 49*.*1)*	*35*.*7*	*(33*.*9 to 37*.*6)*	*34*.*9*	*(33*.*1 to 36*.*8)*
**Continuous**						
per SD increase of FVIII:C^f^	-0.51	(-0.73 to -0.28)	-0.02	(-0.22 to 0.19)	0.15	(-0.06 to 0.37)
**DSST scores (90 points maximum)**						
	**β**_**1**_	**95% CI**	**β**_**2**_	**95% CI**	**β**_**3**_	**95% CI**
**FVIII:C percentile groups**						
≤ p25	1.33	(0.49 to 2.17)	0.50	(-0.21 to 1.21)	0.40	(-0.32 to 1.13)
p25-p75	0	ref	0	ref	0	ref
> p75	-1.87	(-2.73 to -1.01)	-0.69	(-1.42 to 0.04)	-0.22	(-0.97 to 0.53)
***Model variance***	*119*.*5*	*(114*.*1 to 125*.*1)*	*83*.*4*	*(79*.*5 to 87*.*4)*	*80*.*3*	*(76*.*5 to 84*.*3)*
**Continuous**						
per SD increase of FVIII:C	-1.11	(-1.46 to -0.76)	-0.37	(-0.67 to -0.06)	-0.11	(-0.43 to 0.22)

Abbreviations: FVIII:C = coagulation factor VIII activity; 3MSE = Modified mini-mental state examination; DSST = Digit Symbol Substitution Test; CI = confidence interval; p25 = 25th-percentile; p75 = 75th-percentile; ref = reference category; SD = standard deviation.

β coefficients were calculated using linear mixed-effects regression with random intercepts in three models.

^a^ percentage of normal pooled plasma

^b^ unadjusted model

^c^ model adjusted for demographic risk factors (age, gender, ethnicity, education level)

^d^ model additionally adjusted for cardiovascular risk factors (hypertension, smoking status, diabetes, alcohol use, BMI, HDL cholesterol, LDL cholesterol, fibrinogen, log-transformed C-reactive protein, log-transformed maximum common carotid intima-media thickness, log-transformed maximum internal carotid intima-media thickness, and the occurrence of stroke or TIA during follow-up).

^e^ variance between individuals for each model for the primary exposure categorization (percentile groups)

^f^ one standard deviation increase in FVIII:C corresponds to 36.3 units

As a sensitivity analysis, we repeated the above analyses with p10/p90 cutoffs, which did not substantially alter the results. Furthermore, omitting the variable ‘occurrence of a TIA or stroke event during the follow-up’ from the third model (as it may be both an intermediate and confounder) resulted in nearly identical point estimates.

## Discussion

In the present study, we estimated the effects of FVIII:C on WMH burden and cognitive function both cross-sectionally and over time in a population-based sample of older persons in the CHS. Although participants with high WMG on the first MRI scan had higher mean FVIII:C than participants with no or low WMG, no strong association was observed between high FVIII:C and WMH burden in the cross-sectional analyses. Furthermore, high FVIII:C did not appear to be linked to worsening of the WMG between MRI scans performed about 5 years apart after adjustment for confounding variables.

We additionally investigated the relationship between high FVIII:C and cognitive ability as measured by two different scores. Although crude analyses showed an association, no meaningful influence of high FVIII:C on 3MSE scores was observed in cross-sectional analyses after adjusting for potential sources of confounding [27–30]. FVIII:C showed a small association with DSST scores after full adjustment; on average, low FVIII:C (≤p25) levels were associated with scores 0.85 points higher (95%CI 0.06 to 1.64) and high levels (>p75) were associated with scores 0.69 points lower (95%CI -1.52 to 0.13) than the reference group (p25-p75). A dose response was observed across FVIII:C quintile groups and DSST scores in crude models, though the effect sizes attenuated with adjustment. No meaningful relationship was observed between high FVIII:C at baseline and cognitive worsening over time as measured by the 3MSE or DSST after full confounding adjustment.

Although the relationship between WMH and cognitive outcomes is well described in the literature [2–6], few studies have investigated the potential relationship between FVIII:C levels and WMH. An earlier CHS paper with exploratory aims included FVIII:C as one of 70 potential predictors for WMH worsening [22]. However, these exploratory analyses were distinct from the goals and corresponding approaches in our paper, as we aimed to obtain a confounding-adjusted estimates of the specific effect of FVIII:C on WMG as well as FVIII:C on cognitive function. In the present study, potential sources of confounding were selected *a priori*, and the methodology was not dependent on the combined explained variance of a regression model.

Although not in line with our original hypotheses, these results do align with some findings from previous publications. For example, results from the ARIC study indicated that FVIII might have another role in brain morphology that is not specifically related to white matter lesion development; for instance, multiple midlife systemic inflammatory markers (including FVIII) were associated with brain volume late in life [31]. Another recent publication from the same cohort examined the relationship between an “inflammation composite score” that included factor VIII in middle-aged individuals and 20-year cognitive decline [32]. The authors found that each SD increase in the score was associated with additional decline, and that the highest scores were associated with steeper declines over the 20-year period [32]. Taken together, it is feasible that FVIII exerts an effect over a longer period than we captured in our study on cognitive function and by other pathways than via WMH development.

Results from the REGARDS study showed an association between high FVIII levels and cognitive impairment in crude analyses, which were attenuated after multivariable adjustment, similar to our findings [33,34]. These findings may indicate that high FVIII is a marker of one or more underlying processes that play a causal role in cognitive impairment with increasing age, but is perhaps itself not a cause. Although our study did not investigate dementia diagnosis as an outcome, an earlier prospective study of middle-aged men found an association between factor VIII levels and other coagulation markers on vascular dementia after 17 years of follow-up (HR, 1.79; 95% CI, 1.09–3.00) [11]. More recently, a systematic review and meta-analysis synthesized findings from 32 identified studies on the role of the hemostatic system (including some coagulation factors) in cognitive decline in older persons. Among individuals with vascular dementia, higher levels of FVIII were observed together with other coagulation factors [35].

### Study strengths and limitations

Strengths of this study include its population-based, prospective design, large number of older participants, and multiple outcomes and time points of their assessment. Information about many potential sources of confounding was collected and very few values were missing at baseline (<2% of all potential confounding variables). Furthermore, starting with the sixth follow-up, telephone-based estimates could be used to estimate missing 3MSE scores when in-person visits were not possible.

While we were able to control for confounding by including a larger number of sociodemographic and cardiovascular risk factors, we acknowledge that some residual confounding may still be present. However, adjusting for well-known, potentially strong sources of confounding, such as age, did not change our results materially, suggesting that confounding due to weaker unmeasured sources would not have substantially altered our results. It is also possible that Model 3 resulted in some overadjustment, as the occurrence of a stroke or TIA during follow-up could be both a source of time-dependent confounding and an intermediate on the causal path.

Our sensitivity analysis revealed only small differences in the point estimates when this variable was removed from the model, which would not change our interpretation of the results.

Readers should also consider some limitations with respect to the FVIII:C measurements when interpreting our findings. In addition to its implication in thrombosis, FVIII is also a known marker of relevant biological mechanisms such as inflammation and is an acute phase reactant. Though the measurements in the CHS were taken when participants were healthy at baseline, subclinical disease cannot be ruled out. The laboratory measurements of FVIII:C were somewhat variable in the CHS study (coefficients of variation 9% and 10%), however, we do not anticipate this variability in the laboratory measurements had any meaningful impact on our results with such a large sample size. Furthermore, in this secondary data analysis, only one FVIII:C measurement (taken at baseline) was available. When possible, future studies on this topic should consider repeating these measurements to better understand how changes in FVIII:C over time may impact WML and cognitive test performance in the healthy general population.

With respect to our outcome assessments, given the non-linear nature of the WML grading scale, we chose *a priori* to perform ordinal analyses to use all ordered information captured by WMG. However, due to small group size in some WMG categories, which was anticipated among the older general population, we collapsed the higher categories, thereby reducing the resolution of the data. Furthermore, for the longitudinal analyses of worsening WMG, only survivors remaining in the study through the eighth follow-up could be included due to the need for the follow-up MRI, a selection that inherently leads to selection bias. As shown previously, participants who underwent at least one MRI scan were healthier than those who were never scanned, and those with both the initial and follow-up scans were healthier than those who only had the initial MRI scan [17], however, this is a limitation common to all studies involving MRI, and part of our motivation to also look at cognitive function measures. Future studies with more frequent scans over a longer time period may provide valuable insights, however, feasibility of such studies is remains a large obstacle.

We emphasize that these findings from an older, general population cohort should not be extrapolated to younger individuals or specific chronically ill populations. We also cannot rule out that the weak cross-sectional associations observed between FVIII:C and WMG may be explained by reverse causation. WML, as an inflammatory stimulus, could also be a contributing cause of higher FVIII:C levels.

With regard to the cognition outcomes, we acknowledge that the true relationship between high FVIII:C and 3MSE scores in both cross-sectional and longitudinal analyses may be stronger than what we observed due to the ceiling effect of the 3MSE and the high number of participants with high scores. However, upon comparing these results with a second measurement of cognitive function, the DSST (without a ceiling effect), we observed no material differences. We did not adjust any of our longitudinal models for baseline cognitive test scores, as this procedure introduces bias under some circumstances [26]. It is well known that individuals with lower cognitive function are less likely to remain in studies, therefore, attrition was likely differential with regard to the outcomes in the analyses using cognitive test scores. Therefore, the cognitive decline of study participants is likely greater than what we observed in the participants who remained in the study. Although we had two measures of cognitive function, we further acknowledge that neither assesses any one cognitive domain in depth, and readers should interpret our results with this limitation in mind.

## Conclusions

Our findings from this large, population-based study indicate no strong relationship between FVIII:C and WMH burden or WMH worsening over time in the older general population following adjustment for demographic and cardiovascular factors. Furthermore, FVIII:C levels do not appear to be associated with cognitive worsening, as measured using serial 3MSE and DSST assessments. Though an important player in hemostatic balance and implicated in overt vascular disease, this study suggests that FVIII:C is not strongly related to covert, subclinical brain lesion development, and subsequent cognitive decline in older adults.
